# Invasive enterococcal infections in Poland: the current epidemiological situation

**DOI:** 10.1007/s10096-016-2607-y

**Published:** 2016-03-05

**Authors:** I. Gawryszewska, D. Żabicka, K. Bojarska, K. Malinowska, W. Hryniewicz, E. Sadowy

**Affiliations:** Department of Molecular Microbiology, National Medicines Institute, ul. Chełmska 30/34, 00-725 Warsaw, Poland; Department of Epidemiology and Clinical Microbiology, National Medicines Institute, ul. Chełmska 30/34, 00-725 Warsaw, Poland

## Abstract

The aim of this study was to investigate human invasive isolates of enterococci, obtained through prospective surveillance in Poland. The consecutive enterococcal isolates were collected in 30 hospitals between May 2010 and June 2011, and studied by species identification, antimicrobial susceptibility testing and, for *Enterococcus faecium* by detection of markers specific for the hospital meroclone, multilocus VNTR analysis (MLVA) and multilocus sequence typing (MLST). Additionally, the genomic difference regions (GDRs) characteristic for lineage 78 were searched by PCR. Among 259 isolates, a nearly equal number of *Enterococcus faecalis* (*n* = 140; 54.1 %) and *E. faecium* (*n* = 112; 43.2 %) was found. The observed 14-day mortality rate of infected patients reached 18.1 %. All isolates were susceptible to linezolid and daptomycin. High-level aminoglycoside resistance occurred in over 50 % of isolates. Vancomycin resistance mediated by *vanA* or *vanB* was detected in 7.1 % of *E. faecium*; 71.4 % of isolates were multidrug resistant. *E. faecium* isolates ubiquitously carried molecular markers of hospital-associated meroclone (IS*16*, *esp*_*Efm*_, *intA* of ICE*Efm1*) and multilocus sequence typing showed the domination of representatives of lineages 78 and 17/18 (52.7 % and 46.4 %, respectively). Isolates of lineage 78 were significantly enriched in all the GDRs studied. The recent spread of *E. faecium* from this lineage contributed to the observed increase of *E. faecium* in enterococcal invasive infections in hospitals in Poland.

## Introduction

Bacteria belonging to the genus *Enterococcus* are a part of the normal, harmless faecal flora of humans and animals. However, they may also act as opportunistic pathogens, and today are recognized as important causative agents of both invasive and non-invasive nosocomial infections, affecting immunocompromised, severely ill patients [1]. According to the European Centre for Disease Prevention and Control (ECDC) enterococci are presently third, after *Escherichia coli* and *Staphylococcus aureus,* most frequently isolated bacteria from the healthcare-associated infections (HAIs) in Europe [2]. Currently observed acquisition of resistance to antimicrobials used in the therapy of enterococcal infections is a cause of growing concern [3]. *Enterococcus faecalis* and *Enterococcus faecium* represent two species of the biggest clinical importance, while infections caused by other enterococci are rare [4]. Multilocus sequence typing (MLST) and other typing methods showed the presence of distinct clones associated with hospital infections/outbreaks, named high-risk enterococcal clonal complexes (HiRECCs), within populations of both *E. faecalis* and *E. faecium* [5, 6]. Genomes of isolates belonging to HiRECCs are enriched in mobile genetic elements (MGEs), often associated with genes encoding potential virulence factors and resistance determinants [3, 7]. For *E. faecalis,* two HIRECCs, CC6 (also known as CC2) and CC87, preferentially group hospital clinical isolates [8]. Hospital subpopulation of *E. faecium,* initially described as CC17, was subsequently divided into three lineages, 17, 18, and 78, named after the major sequence type (ST) in each group [9, 10]. Analysis of the MLST data using so-called Bayesian analysis of population structure (BAPS) delimited two groups in the hospital meroclone, 2–1, and 3–3, corresponding to lineages 78 and 17/18 respectively [10]. Acquisition of ampicillin and ciprofloxacin resistance, and the presence of the transferable pathogenicity island (ICE*Efm1*), harbouring the *esp*_*Efm*_ gene, are considered important adaptive features of hospital meroclone of *E. faecium* [11]. Genomic analyses indicated the presence of seven additional regions in the genome of ST203 strain from lineage 78 in comparison to lineage 17/18 [12].

The epidemiology of invasive enterococcal infections in Polish hospitals is monitored by a systematic passive surveillance performed by the National Reference Centre for Susceptibility Testing, located at the National Medicines Institute in Warsaw. However, considering the importance of enterococcal infections, we decided to undertake a study based on enhanced surveillance in selected hospitals during a pre-defined period of time. Objectives of this study were to collect enterococcal isolates from normally sterile body sites together with the relevant clinical data, and to evaluate species distribution and antibiotic susceptibility of these isolates. Additionally, we aimed at more profound phenotypic and molecular characterization of *E. faecium* isolates, as the clinical importance of this enterococcal species is recently increasing in several countries [13], to search for a possible reason of this phenomenon.

## Materials and methods

### Bacterial isolates and phenotypic testing

Consecutive invasive isolates of *Enterococcus* spp. (a single strain per patient from normally sterile body fluids, such as blood, pleural fluid, cerebrospinal fluid, and peritoneal fluid) were collected in 30 collaborating hospitals, located in 26 cities in all regions of Poland, between May 2010 and June 2011. All patient data were collected anonymously in the questionnaire formulated for the purpose of the study. A nosocomial infection was defined as infection which was caused by a strain isolated after 48 hours from admission to the hospital or earlier when patient was transferred from another hospital. Enterococcal isolates were re-identified in the central laboratory by conventional methods, and using the mass spectrometer MALDI Biotyper (Brücker, Bremen, Germany) and Vitek MS (bioMérieux, Marcy l’Etoile, France) for selected isolates. For *Enterococcus hirae*, *Enterococcus durans*, and *Enterococcus avium* identification was conducted additionally with VITEK 2 Compact (bioMérieux). Antimicrobial susceptibility testing for ampicillin, penicillin, imipenem, vancomycin, teicoplanin, gentamicin, streptomycin, linezolid, tigecycline, tetracycline, chloramphenicol, rifampin, ciprofloxacin, quinupristin–dalfopristin and trimethoprim–sulfamethoxazole was performed using the broth microdilution method according to the Clinical and Laboratory Standard Institute guidelines (CLSI), and for daptomycin the Etest method (bioMérieux). The reference strain *E. faecalis* ATCC 29212 was used for quality control. Obtained results were interpreted using available 2015 breakpoints of The European Committee on Antimicrobial Susceptibility Testing (EUCAST) (http://www.eucast.org/) and the CLSI breakpoints for antimicrobials for which the EUCAST breakpoints were not available. Isolates were considered multidrug-resistant (MDR) when they showed resistance to three or more classes of antimicrobials tested [14]. Biofilm formation was evaluated by the quantitative adherence assay in Trypticase Soy Broth with 0.25 % glucose [15], and isolates were classified as biofilm non-producers, weak and strong producers, based on the results of staining with crystal violet [16].

### DNA isolation, gene detection, molecular typing and data analysis

Total DNA was isolated using the Genomic DNA Prep Plus kit according to the manufacturer’s instructions (A&A Biotechnology, Gdynia, Poland). Detection of *esp*_*Efm*_ and IS*16* in *E. faecium* was performed by PCR as previously described [17, 18]. The *intA*_ICE*Efm1*_ gene, encoding the integrase of ICE*Efm1* was detected by PCR with primers designed in this study: intA_up2 (5′-AATTGATTCGATAGTTTAGGTA-3') and intA_dn2 (5′-AATCACTTGCTTACTCTTCAT -3′). *E. faecium* isolates positive for IS*16, esp*_*Efm*_ and *intA* from our laboratory collection [19] served as positive controls. Vancomycin nonsusceptibility determinants *vanA*, *vanB*, and *vanC* were detected as previously described [20, 21], with *E. faecium* BM4147, *E. faecalis* V583, and *Enterococcus gallinarum* BM4147 used as respective positive controls. *E. faecium* isolates were analysed by multiple-locus variable-number tandem repeat (VNTR) analysis (MLVA) according to Top et al. [22] and MLST [5], using the MLST database (http://pubmlst.org/efaecium/) to determine allele numbers and STs (21st April 2015, date accessed). New alleles and allelic profiles were submitted to the above database. MLST data were analyzed with the comparative eBURST analysis against the whole *E. faecium* database (http://eburst.mlst.net/; 21st April 2015, date accessed). Genomic difference regions (named herein GDRs), differentiating lineages 78 and 17/18 [12], were detected by PCR using primers specific for genes located in these GDRs, based on available genomic sequences (GenBank Accession number NC_021994; Table 1). Differences in distributions were assessed using the chi-squared test with *p* value ≤ 0.05 considered significant. Antibiotic susceptibility data analysis with the 95 % confidence intervals for the calculation of resistance ratio was done using the WHONET (http://www.whonet.org).

**Table 1:** Primers targeting the genomic difference regions

Primer	Sequence 5′- > 3′	Gene in AUS0085 (ST203)	Gene product	PCR product size
Efm_R1_up	AATCGATGACGTGGAAGAAGG	Ef_aus00245	cadmium_translocating P-type ATPase	411 bp
Efm_R1_dn	GACTAAAGCGCCAGGACAAC			
Efm_R2_up	ATGTTGCCCAAAAGACGAACC	Ef_aus 01495	riboflavin biosynthesis protein RibD	153 bp
Efm_R2_dn	GGAACGGCTAAAACAAGAAGC			
Efm_R3_up	GCGTGATTTCGGTAATTGGTG	Ef_aus 02036	putative phosphosugar isomerase/binding protein	316 bp
Efm_R3_dn	ATGGGAATAGACCAGGAGCA			
Efm_R5_up	CGTGCGTTCCTTTTTCTACC	Ef_aus 02504	bacteriocin-like protein EntT	368 bp
Efm_R5_dn	GGTTTAGATAGCCCACCAAG			
Efm_R6_up	CCCATGAATCCTGTTGGTTC	Ef_aus 02768	PTS system, lactose/cellobiose-specific IIC component	182 bp
Efm_R6_dn	GCAAAAGTAGCAGGAAGGAC			
Efm_R7_up	TCAGCAAATGATGGCGATACG	Ef_aus 02778	glycosyl hydrolase family 38 protein	374 bp
Efm_R7_dn	ACCAATTCGGAGGAATGACATC			

## Results

### Enterococcal species

Altogether, 259 invasive enterococcal isolates were obtained during the collection period. For 247 (95.4 %) of them, the identification by MALDI Biotyper was consistent with the results obtained with conventional methods and Vitek 2 Compact. For the remaining 12 isolates, additional identification with Vitek MS was used, and its results were in the agreement with the results from MALDI Biotyper in three cases (one isolate of *E. faecalis*, *E. faecium* and *E. durans* each), while in nine cases identification with the Vitek MS confirmed the results of conventional methods (for three *E. faecalis*, five *E. faecium* and one *E. casseliflavus*). Thus, collected isolates comprised 140 strains of *E. faecalis* (54.1 %), 112 of *E. faecium* (43.2 %) and seven isolates (2.7 %) of other *Enterococcus* spp.: three *E. gallinarum*, two E*. durans*, and single representatives of *E. avium* and *E. casseliflavus.*

### Hospitals involved in the study and patients’ data

**Fig. 1:**
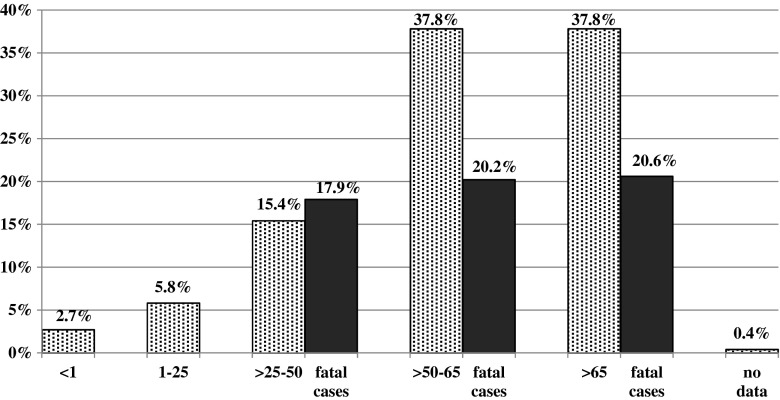
The age distribution of patients and percentage of fatal cases in each age group

Isolates were obtained from 30 collaborating hospitals (8.6 isolates per hospital). Hospitals involved in the study included secondary and tertiary hospitals, mainly of regional coverage (17 provincial hospitals), but also district hospitals (*n* = 5), specialist (*n* = 4) and university (*n* = 4) hospitals. The numbers of isolates collected from each type of ward are presented in Table 2. For five strains, wards of hospitalization were not given. A significant proportion of enterococcal invasive infections were nosocomial infections, i.e., 65.7 % and 78.6 % of *E. faecalis* and *E. faecium* infections respectively. One hundred and forty-five patients (56.0 %) with enterococcal invasive infections were males and 109 (42.1 %) were females; the gender of five patients (1.9 %) was not reported. The age of patients’ ranged from a newborn to 89 years, and the vast majority of isolates (196, i.e., 75.6 %) was obtained from patients aged above 50 years (Fig. 1). The most frequently reported types of infection were bloodstream infections (Table 2), including bacteraemia, septicaemia, and endocarditis (82.9 % of infections caused by *E. faecalis* and 75.0 % infections caused by *E. faecium*) followed by abdominal infections (mainly peritonitis), meningitis, and chest and pelvic infections (mainly abscess). Most of the bacteraemia/septicaemia cases had a known focus (*E. faecalis* 59.3 %, *E. faecium* 53.6 %). The ratio of bloodstream infections to abdominal infections for *E. faecalis* was 6.4:1 and for *E. faecium* 3.8:1, and the observed differences in the ratio of bloodstream infections was statistically significant (*p* = 0.011).

**Table 2:** Ward type, type of infection and outcome reported for patients with invasive enterococcal infections

	*E. faecalis* (140)	*E. faecium* (112)	Other species (7)	All
Number of isolates	140 (54.1 %)	112 (43.2 %)	7	259
Type of ward				
Surgery	32 (22.9 %)	31 (27.7 %)	3	66 (25.5 %)
Haematology/oncology	20 (14.3 %)	32 (28.6 %)	2	54 (20.8 %)
ICU	29 (20.7 %)	27 (24.1 %)	1	57 (22.0 %)
Internal medicine	29 (20.7 %)	11 (9.8 %)	0	40 (15.4 %)
Other neurology (*n* = 9), dialysis centres (*n* = 8), neonatal (*n* = 5), gynaecology (*n* = 4), urology (*n* = 5), infectious disease (*n* = 3), geriatric (*n* = 2), palliative medicine (*n* = 1)	30 (21.4 %)	11 (9.8 %)	1	42 (16.2 %)
Type of infection				
Bacteraemia, septicaemia, including:	109 (77.8 %)	82 (73.2 %)	4	195 (75.3 %)
Bacteraemia	76 (54.3 %)	63 (56.2 %)	3	142 (54.8 %)
Septicaemia	33 (23.5 %)	19 (16.9 %)	1	53 (20.5 %)
- source abdominal infection	16 (11.4 %)	11 (9.8 %)	2	29 (11.2 %)
- source urinary tract infection	10 (7.1 %)	1 (0.9 %)	0	11 (4.2 %)
- other known source	26 (18.6 %)	18 (16.1 %)	0	44 (17.0 %)
- unknown source	57 (40.7 %)	52 (46.4 %)	0	109 (42.1 %)
Endocarditis	7 (5.1 %)	2 (1.8 %)	0	9 (3.5 %)
Abdominal infections	18 (12.9 %)	22 (19.6 %)	3	43 (16.6 %)
Pleural infections	2 (1.4 %)	2 (1.8 %)	0	4 (1.5 %)
Meningitis	2 (1.4 %)	3 (2.7 %)	0	5 (1.9 %)
Pelvic infections	2 (1.4 %)	1 (0.9 %)	0	3 (1.2 %)
Outcome^a^				
All reported	130	107	6	243
Fatal cases	20 (15.4 %)	23 (21.5 %)	1^b^	44 (18.1 %)
Cured	33 (25.4 %)	25 (23.4 %)	2	60 (24.7 %)
Under treatment	77 (59.2 %)	59 (55.1 %)	3	139 (57.2 %)

^a^% of all cases with the known outcome; ^b^ *E. gallinarum*

In the column for each species the % values were calculated separately taking the number of the isolates belonging the each species as 100 %

The data concerning risk factors were available for 165 (63.7 %) of patients, and indicated special importance of previous hospitalization during the previous 6 months (*n* = 124; 75.2 % cases), surgery (*n* = 42; 25.4 %) and an ICU stay (*n* = 35; 21.2 %). Only one patient was reported as a nursing home resident. The reported co-morbidities were: malignancy (*n* = 21; 13.9 %), chronic renal disease (*n* = 7; 4.6 %), diabetes (*n* = 4; 2.6 %), and injury (*n* = 5; 3.3 %). The outcome of treatment of patients with enterococcal infections was reported for 243 cases (93.8 %). Due to the persistence of infection, 2 weeks after strain isolation more than half of the patients (53.7 %) were still receiving antimicrobial treatment, while only 23.2 % of patients were successfully cured. The observed overall 14-day mortality rate reached 18.1 % (44 patients; Table 2). Fatal cases were reported in patients aged above 25 years, and mortality showed an increase with age (Fig. 1). The highest mortality ratio was reported for meningitis (one of three) and septicaemia (30.2 %, 16 of 53). A higher mortality in *E. faecium* infections (23 cases, 21.5 %) than these caused by *E. faecalis* (20 cases, 15.4 %) was observed, however, without statistical significance (*p* = 0.22). A single case of fatal septicaemia in an oncology patient infected with *E. gallinarum* was reported (Table 2). In two cases, VREm infections proved fatal (28.6 % mortality, i.e., two out of seven cases with the reported outcome), while for vancomycin-susceptible strains this value was 21.0 % (21 of 100 with the reported outcome).

### Antimicrobial susceptibility of isolates and biofilm production by *E. faecium*

All isolates were susceptible to linezolid and daptomycin (Table 3), and all *E. faecalis* isolates were also susceptible to ampicillin, vancomycin and teicoplanin. Over 50 % of isolates of both species showed high-level resistance to aminoglycosides. Among *E. faecium* isolates, very high ratios of resistance to ampicillin, ciprofloxacin, and rifampin were observed, and vancomycin resistance was detected in eight isolates (VREfm); five of them showed VanA phenotype and the presence of *vanA*. The three remaining VREfm carried the *vanB* gene. Altogether, 71.4 % of isolates were classified as multidrug resistant (MDR) [14]. This phenotype was specific for nearly all isolates of *E. faecium* (109 isolates, 97.3 %) and 73 isolates (52.1 %) of *E. faecalis*. Only four among 112 isolates of *E. faecium* (3.6 %) were identified as biofilm producers, including three strong producers and a single weak producer.

**Table 3:** Antimicrobial susceptibility profiles of enterococcal isolates from invasive infections

Species	MIC (mg/L)			Number of isolates (%) and 95 % confidence intervals of % R^a^			
Antimicrobial agent^b^	Range	MIC_50_	MIC_90_	S	I^c^	R	95 % CI
*E. faecalis* (*n* = 140)							
Ampicillin	0.5–4.0	2.0	4.0	140 (100 %)	0	0	0-3.3
Penicillin	0.125–16.0	2.0	4.0	139 (99.3 %)	-	1 (0.7 %)	0-4.5
Vancomycin	0.125–4.0	2.0	4.0	140 (100 %)	-	0	0-3.3
Teicoplanin	0.125–0.5	0.125	0.25	140 (100 %)	-	0	0-3.3
HLGR	4.0– >1024	1024	>1024	62 (44.29 %)	-	78 (55.71 %)	47.1-64.0
HLSR	16.0– >2048	2048	>2048	64 (45.71 %)	-	76 (54.29 %)	45.7-62.7
Tetracycline	0.125– >256	128	256	14 (10 %)	2 (1.43 %)	124 (88.57 %)	81.9-93.1
Tigecycline	0.015–0.25	0.062	0.25	140 (100 %)	0	0	0-3.3
Chloramphenicol	0.5–128	8.0	128	97 (69.29 %)	1 (0.71 %)	42 (30 %)	22.7-38.4
Daptomycin	0.125–4.0	1.0	2.0	140 (100 %)	-	0	0-3.3
Rifampin	0.25–16.0	2.0	8.0	45 (32.14 %)	37 (26.43 %)	58 (41.43 %)	32.6-49.3
Ciprofloxacin	0.125– >32.0	8.0	>32	56 (40 %)	13 (9.29 %)	71 (50.71 %)	41.5-58.5
Linezolid	0.25–4.0	2.0	4.0	140 (100 %)	-	0	0-3.3
Imipenem	0.5-64	16	64	11 (7.9 %)	30 (21.4 %)	99 (70.7 %)	62.3-77.9
Trimethoprim-sulphamethoxazole	0.015-32	4	32	11 (7.9 %)	47 (33.8 %)	81 (58.3 %)	49.6-66.5
*E. faecium* (*n* = 112)							
Ampicillin	1– >256	64	128	1 (0.89 %)	11 (9.82 %)	110 (89.29 %)	81.7-94.1
Penicillin	0.25– >256	128	256	9 (8.04 %)	-	103 (91.96 %)	84.9-96.1
Vancomycin	0.5– >256	2.0	4.0	104 (92.86 %)	-	8 (7.14 %)	3.3-14.0
Teicoplanin	0.125–128	0.25	1.0	107 (95.54 %)	-	5 (4.46 %)	1.7-10.7
HLGR	4.0– >1024	>1024	>1024	15 (13.39 %)	-	97 (86.61 %)	78.6-92.1
HLSR	4.0– >2048	2048	>2048	16 (14.29 %)	-	96 (85.71 %)	77.5-91.4
Tetracycline	0.25–256	16	128	49 (43.75 %)	4 (3.57 %)	59 (52.68 %)	43.1-62.1
Tigecycline	0.015–0.5	0.062	0.25	111 (99.11 %)	1 (0.89 %)	0	0-4.1
Chloramphenicol	0.062–32.0	8.0	16	89 (79.46 %)	16 (14.29 %)	7 (6.25 %)	2.7-12.8
Daptomycin	0.062–4.0	2.0	4.0	112 (100 %)	0	0	0.0-4.1
Rifampin	0.062– >128	32	>128	5 (4.46 %)	3 (2.68 %)	104 (92.86 %)	86-96.7
Ciprofloxacin	2.0– >256	256	>256	0	1 (0.89 %)	111 (99.11 %)	94.4-100
Linezolid	1.0–4.0	2.0	4.0	112 (100 %)	0	0	0.0-4.1
Quinupristin-dalfopristin	0.125–16	2.0	4.0	54 (48.2 %)	52 (46.4 %)	6 (%54 %)	2.2-11.8
Other *Enterococcus* spp. (*n* = 7)							
Ampicillin	0.0625–128.0	16	128	3	0	4	-
Penicillin	0.5–64.0	8	64	6	-	1	-
Vancomycin	0.5–8.0	2	8	4	-	3	-
Teicoplanin	0.125–1.0	0.25	1	7	-	0	-
HLGR	4.0–1024	64	1024	5	-	2	-
HLSR	32.0–2048	256	2048	4	-	3	-
Tetracycline	0.125–64	64	64	2	0	5	-
Tigecycline	0.031–0.062	0.031	0.062	7	0	0	-
Chloramphenicol	0.25–8.0	4	8	7	0	0	-
Daptomycin	0.25–2.0	1	2	7	0	0	-
Rifampin	0.062–64.0	0.5	64	5	0	2	-
Ciprofloxacin	0.125–64.0	4	64	3	0	4	-
Linezolid	0.015–4.0	2	4	7	0	0	-

^a^S, susceptible; I, intermediate; R, resistant; ^b^HLGR, high-level gentamicin resistance, HLSR, high-level streptomycin resistance; ^c^ -, intermediate category not defined for this compound

### Molecular typing of *E. faecium* isolates and the distribution meroclone- and lineage-specific markers

MLVA revealed 12 MLVA types (MTs; Table 4) among 112 invasive *E. faecium* isolates, with the most prevalent being MT159, characteristic for 52 isolates (43.3 %) from 20 hospitals. Other frequently encountered MTs included 1, 11, and 12. The subsequent MLST analysis, performed for a group of 46 representative isolates, revealed the presence of 14 STs (Table 4). The comparative eBURST analysis included four STs (78, 192, 341, 412) in lineage 78 and nine STs (17, 18, 64, 80, 117, 202, 262, 877, 878) in lineage 17/18; the remaining ST879 represented a singleton. Isolates of lineage 17/18 were most often associated with MT1 and MT12, and isolates of lineage 78 typically had MT159, however, one ST117 isolate (lineage 17/18) had MT159 (verified by repeated typing). The acquisition of vancomycin-resistance determinants *vanA* and *vanB* occurred chiefly among isolates of lineage 17/18 (five out of seven cases). Most of the isolates able to produce biofilm (three out of four) also belonged to this lineage. IS*16* and *esp*_*Efm*_ were detected in all *E. faecium* isolates, and *intA*_ICE*Efm1*_ for 107 isolates (95.5 %). Thus, the *esp*_*Efm*_ gene (verified by sequencing) was present in five isolates negative for *intA*_ICE*Efm1*_. Distribution of genes located within six GDRs characteristic for ST203 from lineage 78 [12] was assessed among isolates of *E. faecium* collected in this study and for comparative purposes among 52 sewage isolates, not associated with the hospital meroclone [23]. Significant differences in the prevalence were found for GDR1, which was more frequently detected in sewage isolates, and GDR2 and GDR3, which occurred chiefly among hospital-associated isolates (Table 5). Comparison between two hospital lineages of *E. faecium* revealed a significant over-representation of all six GDRs in lineage 78 in comparison to lineage 17/18. Sequencing of PCR products for a few randomly-selected representatives of both lineages and non-hospital *E. faecium* for all six GDRs revealed their 100 % identity with the counterparts in ST203 [12].

**Table 4:** MLVA and MLST of invasive *E. faecium* isolates

MT^a^	VNTR profile^b^	Number of isolates	Number of hospitals	Number of VRE	Number of fatal cases	Number of isolates analysed by MLST	STs^a^	Lineage
1	5-7-3-3-2-3	16	10	3	3	8	17; 18; 64; 80; 202	17/18
7	5-7-3-3-2-2	1	1	0	0	1	17	17/18
10	5-7-3-3-3-3	1	1	0	0	1	262	17/18
11	6-7-3-3-2-3	13	8	0	1	3	202	17/18
12	5-7-3-3-1-3	15	8	2	4	6	877; 117	17/18
112	3-7-4-2-1-3	1	1	0	0	1	878	17/18
159	5-7-3-3-1-2	1	1	0	0	1	117	17/18
520	2-7-3-3-1-2	4	4	1	1	2	117	17/18
	summary	52	21	6	9	23		17/18
159	5-7-3-3-1-2	51	20	2	11	12	78; 192; 341; 412	78
291	5-7-4-3-1-2	4	2	0	1	1	78	78
334	5-7-3-4-1-2	3	1	0	2	1	78	78
518	5-2-3-3-1-2	1	1	0	0	1	78	78
	summary	59	21	2	14	15		78
519	3-7-3-3-2-2	1	1	0	0	1	879	singleton

^a^novel MTs and STs underlined; ^b^in the order: VNTR1, VNTR2, VNTR7, VNTR8, VNTR9, VNTR10

**Table 5:** Distribution of GDRs among lineages 17/18 and 78 and among hospital and sewage isolates of *E. faecium*

lineage^a^/origin	GDR1 (%)	GDR2 (%)	GDR3 (%)	GDR5 (%)	GDR6 (%)	GDR7 (%)	Number of isolates with *n* GDR loci						
							*n* = 0	*n* = 1	*n* = 2	*n* = 3	*n* = 4	*n* = 5	*n* = 6
17/18 (*n* = 27)	14 (51.2)	16 (59.2)	5 (18.5)	4 (14.8)	6 (22.2)	2 (7.4)	4	12	4	3	3	0	1^b^
78 (*n* = 18)	16 (88.9)	18 (100)	15 (83.3)	18 (100)	11 (61.1)	13 (72.2)	0	0	0	2	3	5	8
*P* ^c^	0.02	0.005	0.0001	0.00000	0.02	0.00003							
ST879 (*n* = 1)	1	0	1	1	0	1					1		
Hospital (*n* = 46)	31 (67.4)	34 (73.9)	21 (45.6)	23 (50.0)	17 (36.9)	16 (34.8)	4	12	4	5	7	5	9
Sewage (*n* = 52)	49 (94.2)	0 (0)	5 (9.6)	21 (40.4)	11 (21.1)	11 (21.1)	2	20	13	16	1	0	0
*P* ^d^	0.001	0	0.0001	0.4	0.1	0.2							

^a^Based on the MLST data; ^b^verified by repeated typing and GDR sequencing; ^c^differences in GDR distributions between lineages 17/18 and 78 isolates; ^d^differences in GDR distributions between hospital and sewage isolates

## Discussion

The growing importance of enterococcal infections in hospitals prompted us to perform an enhanced surveillance of enterococci causing invasive diseases in selected Polish hospitals in the pre-defined period of time. The clinical presentations of infections were typical for enterococci, including mainly bacteraemia and abdominal infections. Similarly to other reports, our results point out the abdominal infections as the most frequently observed sources of bacteraemia/septicaemia; however, the small proportion of *E. faecalis* bacteraemia originating from urinary tract (7.1 %) was surprising [24, 25]. This could be due to the relatively high ratio of bacteraemia with unknown source observed in our study for both *E. faecalis* and *E. faecium* (40.7 % and 46.4 % respectively) in comparison to other reports (*E. faecalis* 30 % and 21.6 %; *E. faecium* 39.9 % and 20.7 %) [24, 25]. Collected clinical data confirmed established risk factors for acquisition of invasive enterococcal infection, such as advanced age, previous hospitalization, and ICU stay [13, 24]. In our study, the majority of patients were male and aged above 50 years, and most of the fatal cases were observed in this age group. Other studies have reported similar proportions of patients’ gender and age distribution [24]. The 14-day mortality rate of 18.1 % reported in our study corresponded to the 30-day mortality rates of 18.9 % to 25 % among patients with enterococcal bacteraemia in other countries [25]; however, it is important to note that at the 2-week reporting interval the majority of patients in our study were still under treatment. We observed a higher mortality in infections caused by *E. faecium* than *E. faecalis* (20.1 % vs 15.4 %), which is in agreement with a report from Denmark (34.6 % and 21.4 % respectively) [24] and Spain (30 % and 26 % respectively, among cancer patients) [26].

While at the rise of enterococcal infections in hospitals in the 1970s *E. faecalis* represented the most predominant species, contributing to approximately 90 % of infections, more recently the relative proportion of these two species has been changing in the favour of *E. faecium* and is now almost reaching or even exceeding the parity [26, 27], due to the increasing incidence of infections caused by *E. faecium* [24]. This worldwide trend of increasing importance of *E. faecium* in HAIs was also observed in the present study, where 54 % and 43 % of infections were caused by *E. faecalis* and *E. faecium* respectively. For comparison, the ratio of *E. faecium* accounted for around 30 % of isolates from blood in our country during 2001–2004, and increased to around 40 % in 2009–2013 (http://www.ecdc.europa.eu/en/activities/surveillance/EARS-Net). This shift in the proportion of *E. faecium* to *E. faecalis* results in the increased overall morbidity of enterococcal infections, due to a higher fatality ratio of bacteraemia caused by this species [26], which may be at least partially associated with frequent resistance of *E. faecium* to important anti-enterococcal drugs, such as ampicillin, aminoglycosides, and glycopeptides [24–26]. Ampicillin resistance is now very common among hospital *E. faecium,* and it proceeded the appearance and constant rise of vancomycin resistance in the hospital meroclone of this species [11, 28]. In our study, acquired resistance to vancomycin in *E. faecium* was still relatively rare (7 %). VREfm have been observed in Poland since the end of the 1990s with an increasing incidence [19, 29]. The frequency of VRE differs in various regions of the world, with a high prevalence reported in the US (up to 80 % of *E. faecium*) [27] and some European countries, such as Greece, the UK, and Portugal [28], while a low ratio (below 1 %) is characteristic for Sweden, The Netherlands, France, and Spain (http://www.ecdc.europa.eu/en/activities/surveillance/EARS-Net). We observed a very high prevalence of high-level resistance to aminoglycosides in *E. faecium*, which eliminates the possibility of combined therapy including aminoglycoside with cell-wall-active agents (penicillins, glycopeptides) for synergistic bactericidal effect. Although *E. faecalis* remains generally susceptible to penicillins, high-level resistance to aminoglycosides exceeded 50 % in our study, with the same problem for the combined therapy. Such a phenotype has also been reported for several other European countries (http://www.ecdc.europa.eu/en/activities/surveillance/EARS-Net), and our previous analysis of nosocomial isolates of *E. faecalis* from six European countries showed a significant role of two hospital clones in the spread of high-level aminoglycoside resistance in this species [8]. All isolates in the current study showed susceptibility to linezolid, the drug of last resort in VRE infections, although resistance to this compound is being observed in our country for both *E. faecalis* and *E. faecium* [30].

Selection of particular enterococcal clones, adapted to hospital settings, appears to play a crucial role in the increasing importance of these pathogens in HAIs, especially in the case of *E. faecium*. The rise in the prevalence of this species which was found in our study prompted us to analyse collected *E. faecium* isolates in more detail. The results of MLVA, MLST and detection of markers, specific for nosocomial *E. faecium* such as IS*16* [18], *esp*_Efm_ and *intA*_ICE*Efm1*_ associated with a transferable pathogenicity island IC*Efm1* [31, 32], indicated that almost all isolates represented hospital epidemic meroclone. Circulation of strains belonging to this clone most likely predates the appearance of the first VRE in Polish hospitals in the late 1990s [19, 29]. Esp_Efm,_ a species-specific variant of the enterococcal surface protein, is involved in biofilm formation [33] and increases enterococcal virulence in endocarditis [34]. Although the *esp*_Efm_ gene was present ubiquitously in the studied group, only a minority of isolates were able to form biofilm under experimental conditions. A recent study [35] questioned the utility of a polystyrene dish assay for biofilm formation by *E. faecalis*, and showed much more reliable performance of porcine heart valve explants. A similar system may also be required for *E. faecium* biofilm studies.

Hospital *E. faecium* in our study belonged to both major BAPS groups, corresponding to lineages 17/18 and 78 [10]. The latter, more recently evolving lineage is currently being isolated in hospitals all over the world [26, 36, 37], and in Poland it was first observed in 2005 among VREfm [19]. ST78 and its variants are typically associated with MT159 [38, 39], the most prevalent MT in our study. Spread of lineage 78 strains is considered to be a significant factor of the increasing VanB-type vancomycin resistance in Australia [37], and current detailed genomic analysis of strains representing both lineages has revealed the presence of seven GDRs, additionally present in a representative of lineage 78 [12]. In this study, we analysed the distribution of genes characteristic for six GDRs, with the exception of one region (GDR4) which contains a presumable integrase gene. Genes of two GDRs, namely the gene encoding a riboflavin biosynthesis protein RibD from GDR2 and the gene encoding a putative phosphosugar isomerase from GDR3, were significantly more prevalent among isolates from hospital settings in comparison to sewage isolates, while the latter were more often carrying the gene of cadmium-translocating P-type ATPase from GDR1; it is likely that the product of this gene provides a selective advantage in various environments. Among hospital strains, all six analysed GDRs were over-represented among successful lineage 78; however, genes of GDR5, 6, and 7 were relatively frequent also among the sewage isolates, suggesting that *E. faecium* circulating in the community might have been a source of such genes for nosocomial strains. Further analyses are, however, required to verify such a possibility.

In summary, our study provides the data on species distribution, prevalence of resistance, and clonality of enterococcal invasive isolates, and characterizes patients affected by such infections. The study on Polish isolates shows the similarity of invasive, hospital-adapted *E. faecium* to strains circulating in other countries, and underlines the importance of permanent surveillance of the dynamic epidemiological situation concerning these dangerous opportunistic pathogens. These data will be the reference for future studies performed in Poland.
